# Production of ascorbic acid releasing biomaterials for pelvic floor repair

**DOI:** 10.1016/j.actbio.2015.10.019

**Published:** 2016-01-01

**Authors:** Naşide Mangır, Anthony J. Bullock, Sabiniano Roman, Nadir Osman, Christopher Chapple, Sheila MacNeil

**Affiliations:** a Department of Materials Science Engineering, Kroto Research Institute, University of Sheffield, United Kingdom; b Royal Hallamshire Hospital, Urology Clinic, Sheffield, United Kingdom

**Keywords:** Extracellular matrix, Collagen, Polylactic acid, Pelvic floor tissue engineering

## Abstract

**Objective:**

An underlying abnormality in collagen turnover is implied in the occurrence of complications and recurrences after mesh augmented pelvic floor repair surgeries. Ascorbic acid is a potent stimulant of collagen synthesis. The aim of this study is to produce ascorbic acid releasing poly-lactic acid (PLA) scaffolds and evaluate them for their effects on extracellular matrix production and the strength of the materials.

**Materials and methods:**

Scaffolds which contained either l-ascorbic acid (AA) and Ascorbate-2-Phosphate (A2P) were produced with emulsion electrospinning. The release of both drugs was measured by UV spectrophotometry. Human dermal fibroblasts were seeded on scaffolds and cultured for 2 weeks. Cell attachment, viability and total collagen production were evaluated as well as mechanical properties.

**Results:**

No significant differences were observed between AA, A2P, Vehicle and PLA scaffolds in terms of fibre diameter and pore size. The encapsulation efficiency and successful release of both AA and A2P were demonstrated. Both AA and A2P containing scaffolds were significantly more hydrophilic and stronger in both dry and wet states compared to PLA scaffolds. Fibroblasts produced more collagen on scaffolds containing either AA or A2P compared to cells grown on control scaffolds.

**Conclusion:**

This study is the first to directly compare the two ascorbic acid derivatives in a tissue engineered scaffold and shows that both AA and A2P releasing electrospun PLA scaffolds increased collagen production of fibroblasts to similar extents but AA scaffolds seemed to be more hydrophilic and stronger compared to A2P scaffolds.

**Statement of significance:**

Mesh augmented surgical repair of the pelvic floor currently relies on non-degradable materials which results in severe complications in some patients. There is an unmet and urgent need for better pelvic floor repair materials. Our current understanding suggests that the ideal material should be able to better integrate into sites of implantation both biologically and mechanically.

The impact of vitamin C on extracellular matrix production is well established but we in this study have undertaken a critical comparison of two derivatives of vitamin C as they are released from a biodegradable scaffold. This strategy proved to be equally useful with both derivatives in terms of new tissue production yet we observed significant differences in mechanical properties of these biomaterials.

## Graphical abstract

**Figure:**
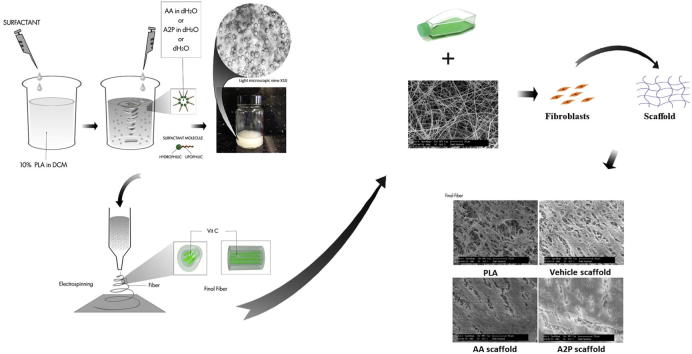

## 1 Introduction

Stress urinary incontinence (SUI) and pelvic organ prolapse (POP) are two major public health issues that can affect the physical, social and psychological wellbeing of affected women, usually aged >40 years [1], [2]. Surgical treatment of both conditions often involves implantation of biomaterials to provide support. Although there is a reasonable initial success rate, 20–30% of women will subsequently require re-operation due to complications or recurrence [3], [4]. The complications related to synthetic non-absorbable mesh implants are particularly concerning for both patients and physicians [5], [6]. Tissue engineering has potential to overcome such complications through the use of degradable synthetic matrices with appropriate strength and elasticity that release bioactive factors conducive to integration of the material and tissue remodelling.

Healthy pelvic floor connective tissues are thought to be in a state of continuous remodelling involving the synthesis and degradation of collagen and other ECM components [7]. Abnormalities in this degradation-synthesis equilibrium are not only implicated in the pathophysiology of pelvic floor defects but may also put any repair at risk of failure as the underlying primary abnormality in collagen turnover persists. Degradation of collagen is mediated by a family of matrix metalloproteinases (MMP), whose activity is regulated at multiple levels including synthesis, activation, activity inhibition (tissue MMP inhibitors) and synthesis of collagen involves a cascade of post-translational modification reactions leading to a mature, high tensile strength support structure. Elevated MMP and reduced MMP inhibitor levels were found in paravaginal tissues of women with urinary incontinence [8] and also reduced collagen content was evident in endopelvic fascia of women with SUI and POP compared to healthy women [9], [10]. Thus defective/decreased collagen synthesis is a part of the disease process in women with SUI and/or POP and incorporation of stimulants of collagen synthesis into biomaterials could be a reasonable strategy in pelvic floor tissue engineering.

Ascorbic acid plays a crucial role in synthesis and post-translational modification of collagen. This is widely studied and thought to occur at multiple levels: [1] acting as a co-factor for the enzyme prolyl hydroxylase which is responsible for crosslinking of collagen fibrils thus forming the triple helix structure [11], [2] stimulating collagen gene expression via malondialdehyde [12] and [3] activation of collagen gene transcription and stabilization of procollagen mRNA [13], [14]. Also ascorbic acid is a major antioxidant in human blood at concentrations of 40–80 μM. Safe intravenous administrations up to a final plasma concentration of 5 mM in cancer patients have been reported [15]. The toxic concentrations in culture conditions are consistently reported to be >0.5 mM [16]. The use of l-ascorbic acid (AA), the naturally occurring and metabolically active form of ascorbic acid in humans, to increase collagen production of fibroblasts *in vitro* dates back to 1970s [17]. Its use is limited by its low chemical stability in aerobic culture conditions [18]. Thus a variety of AA derivatives have been produced to increase its stability (e.g. Ascorbate-2-Phosphate [A2P], Na salt of ascorbate). A2P has been the most widely used synthetic form of AA in cell culture since Hata and Senoo proved the efficacy of the long acting and stable A2P [19], [20]. There have been a few direct comparisons of AA analogues in cultured fibroblasts [21], [22].

Electrospinning is a widely used technique in tissue engineering that produces scaffolds with micro/nano sized fibres while allowing purposeful modification of the 3D structure [23]. Also electrospun PLA scaffolds were shown to have mechanical properties close to those of healthy paravaginal tissue [24] while showing successful integration into native tissues in the short term [25]. Emulsion electrospinning is a modification of this technique to achieve successful incorporation of hydrophilic substances, such as AA, in the centre of hydrophobic polymer fibres resulting in a core–shell morphology that enables a sustained release of the hydrophilic content [26], [27]. Thus the emulsion electrospun mats can serve as both vehicles to deliver bioactive factors and tissue scaffolds to provide structural support [28]. Previously successful encapsulation of vascular endothelial growth factors [29], Rhodamine B [30] and human nerve growth factor [31] into emulsion electrospun fibres has been demonstrated.

The aims of this study were to construct electrospun PLA scaffolds that are able to release the two most commonly used ascorbic acid derivatives (AA and A2P), to evaluate the comparative effectives of the two in terms of collagen production and finally to assess the impact of the AA and A2P on the mechanical properties of the electrospun scaffolds.

## 2 Materials and methods

### 2.1 Scaffold synthesis and characterisation

#### 2.1.1 Preparation of emulsions

**Fig. 1:**
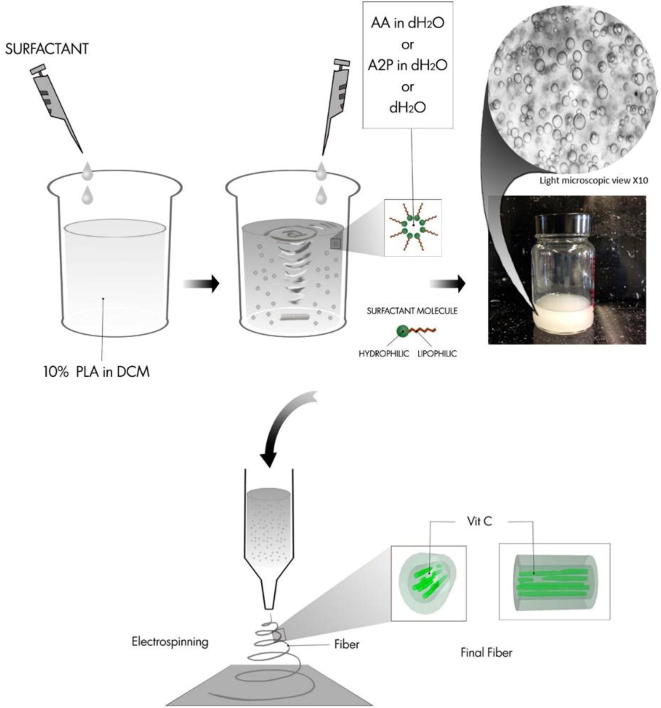
Preparation of the emulsions containing AA, A2P or dH_2_O (Vehicle) and proposed mechanism of how the emulsion electrospinning technique produces a core–shell morphology of hydrophilic (vitamin C) centre out-layered by the hydrophobic PLA (PLA: polylactic acid, DCM: dichloromethane, AA: ascorbic acid, A2P: Ascorbate-2-Phosphate, dH_2_O: distiled water).

PLA polymer (Sigma–Aldrich) was dissolved 10% w/v in dichloromethane (DCM). Fifty microlitre of Span80 (Sigma–Aldrich) was added to the polymer solution and stirred at 250 rpm for 10 min. l-ascorbic acid (Sigma–Aldrich) and l-ascorbic acid 2-phosphate (SigmalAldrich) were dissolved in distiled water and a total volume of 500 μl solution was added drop wise to the PLA-Span80 solution while stirring 1000 rpm with magnets for 15 min (Fig. 1), the final concentration being 0.0001, 0.001 and 0.01 g of either AA or A2P per gram of PLA. Unless stated otherwise, the medium concentration (0.001 g of AA and A2P per gram of PLA) was used in experiments. A control emulsion electrospun scaffold containing only 500 μl dH_2_O (Vehicle scaffolds), without AA or A2P was also included together with a PLA only electrospun scaffold. All emulsions were freshly made and electro spun immediately.

#### 2.1.2 Electrospinning conditions

The emulsions were loaded into 5 mL syringes with blunt tipped stainless steel needles. The emulsions were delivered at a constant feed rate of 40 μl/min using a programmable syringe pump (Aladdin 1000) and were electrospun horizontally with an accelerating voltage of 15 kV supplied by a high voltage power supply (Brandenburg, Alpha series III, UK). Fibrous mats were collected on aluminium foil sheets wrapped around an earthed aluminium rotating collector (rotating at 300 rpm) 15 cm from the tip of the needle. Scaffolds were produced and left to dry for 1 h in a fume hood.

#### 2.1.3 AA and A2P release profile

All measurements of AA and A2P were performed using a UV-spectrophotometer (Thermo Scientific™ Evolution 220) at an absorbance wavelength of 252 nm. A calibration curve was initially constructed by measuring 8 concentrations of AA and A2P (lowest: 10 nM and highest: 100 μM) prepared in dH_2_O and PBS, respectively. All solutions were freshly prepared and the absorbances were immediately measured. The calibration curve was linear with a correlation coefficient of *R*^2^ > 0.999 for both AA and A2P and the lower limit of detection was 500 nM.

**Fig. 2:**
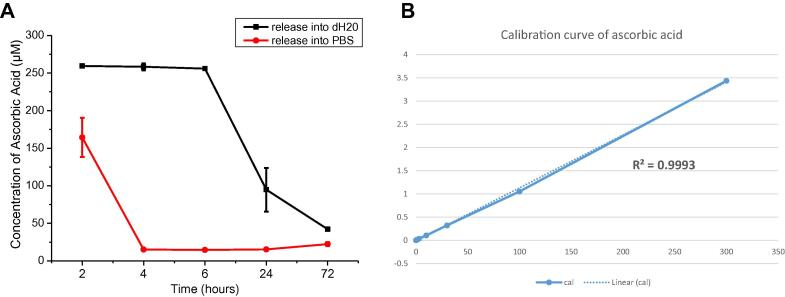
Comparison of dH_2_O and PBS as a media to study AA release (A) and the calibration curve of ascorbic acid as studied in dH_2_O (B).

Initially the feasibility of using PBS to demonstrate AA release was investigated. For this three pieces of AA scaffolds (mean weight 0.022 ± 0.001 g) were placed into 4 mL of dH_2_O or PBS. At 2, 4, 6, 24 and 72 h media was removed, absorbance read and the media was put back into the well. The concentration was determined using the calibration curve. The release of AA into dH_2_O could be demonstrated with small error bars within the first 6 h of the experiment whereas we were unable to detect any amount of AA in PBS except at the first 2 h (Fig. 2). This suggested dH_2_O as the sole media to study release of AA.

Release of AA and A2P from scaffolds were studied in dH_2_O and PBS, respectively. Three pieces of AA and A2P scaffolds (mean weights: 0.0199 ± 0.002 and 0.0176 ± 0.002, respectively) were placed in 4 mL media and were kept in a dry incubator at 37 °C. A vehicle scaffold was taken as a control. At 2, 4, 6, 8, 10 h and 1, 2, 3, 7, 14, 21 and 28 days a sample was removed from the media, the absorbance measured and the concentration was determined with use of the calibration curve. All the media were then discarded and replaced with fresh media. Experiments were repeated three times.

To determine the actual amount of AA or A2P encapsulated within the micro/ nanofibers, an accurately weighed 20 mg piece of scaffold was dissolved in 10 mL of DCM and 5 mL of distiled water was then added. After vortexing vigorously for 15 min samples were left to stand still for an hour to allow phase separation. One millilitre of clear supernatant was then taken and absorbances were read at 252 nm for determination of AA and A2P concentration. The following formula was used to calculate encapsulation efficiency (%):

(Actual drug content/Theoretical drug content)×100.

where actual drug content is the amount drug extracted from the scaffold and theoretical drug content is the total quantity of the drug added while preparing the emulsion for electrospinning.

#### 2.1.4 Water uptake measurement

Water uptake by scaffolds was assessed by incubating weighed 1 × 1 cm pieces of each scaffold in 10 mL of PBS. Six samples for each group were measured. Scaffolds were incubated at 37 °C for 21 days and at days 1, 2, 3, 7, 14 and 21 they were carefully blotted with filter paper to remove surface water and weighed. Water uptake of each scaffold was expressed as the percent increase in weight of the scaffold and calculated with the formula:

$$({\text{Weight}}_{\text{wet}}-{\text{Weight}}_{\text{dry}})/{\text{Weight}}_{\text{dry}}\times 100\text{.}$$

### 2.2 Cell response to scaffolds in 2D and 3D cultures of fibroblasts

#### 2.2.1 Culture conditions of human dermal fibroblasts

Human dermal fibroblasts (HDFs) were isolated from skin obtained from consenting patients undergoing elective surgical procedures as described previously [32]. Cells were isolated and expanded in complete cell culture media consisting of Dulbecco’s Modified Eagles’s Medium (DMEM) (Biosera, UK) supplemented with 10% foetal calf serum, 2 mM l-glutamine, and 1% penicillin/streptomycin. HDFs were incubated at 37 °C in the presence of 5% CO_2_ with fresh media changes every 3–4 days and used between passages 4–9. Each experiment consists of three sample wells for each condition and was performed in triplicates.

For 2D experiments a total of 50,000 HDFs were seeded per well of 12 well plates. Media containing 0, 10, 100, 300 and 600 μM of AA or A2P were prepared freshly and changed every day or every 3–4 days for 14 days. For experiments with confluent fibroblasts, supplementation was started after cells were confluent. The results of collagen production in this section were presented as a percent (%) increase in collagen production compared to collagen production of fibroblasts grown in non-supplemented media. In 3D experiments, 0.79 cm^2^ scaffolds were kept in 70% ethanol for 10 min after which a total of 500,000 HDFs were seeded and cultured for 14 days.

#### 2.2.2 Assessment of cell viability

Cell metabolic activity was measured by a resazurin assay. Briefly at 1, 3, 7 and 14 days of culture all media was removed from culture plates, each well was washed with PBS and 5 mL of 125 μl/mL resazurin in PBS was added. Samples were incubated for 60 min at 37 °C. Then absorbance at 570 nm was measured in a colourimetric plate reader (Bio-TEK, NorthStar Scientific Ltd, Leeds, UK). Culture wells without cells or cell free scaffolds in media were used as reagent blanks. Samples were then washed of the dye and were fixed with 3.7% formaldehyde. Experiments were performed 3 times in triplicate.

#### 2.2.3 Sirius Red staining for total collagen

Fixed samples were washed with PBS. Two millilitre of Sirius Red stain (0.1% Direct Red 80 in saturated picric acid, Sigma–Aldrich) was added to each sample of monolayer cultured fibroblasts or fibroblasts grown on scaffolds. After 16 h, excess stain was washed off with distiled water until the wash out water ran clear. Then specimens were dried, weighed and the stain eluted with 2 mL 0.2 M NaOH:methanol 1:1 for 15 min. The absorbance was measured at 490 nm. Acellular scaffolds acted as controls for calculating collagen production. Results were expressed as Sirius Red stain per gram of PLA.

### 2.3 SEM

The samples were washed in PBS and fixed in 10% buffered formaldehyde solution for 10–15 min. A 0.1 M cacodylate buffer was added and incubated for 20 min, glutaraldehyde in cacodylate buffer was added to the samples for 30 min. Samples were then washed twice for 15 min each wash in 0.1 M cacodylate buffer. Next samples were then incubated for 2 h in osmium tetraoxide. Samples were then incubated in 0.1 M cacodylate buffer for 15 min. Following this, samples were incubated in increasing concentrations of ethanol (75%, 95% and 100%) for 15 min each and were incubated for 30 min in 100% ethanol dried over anhydrous copper sulphate. Finally the ethanol was removed and hexamethlydisalazine was added to the samples for 30 min and the samples were then left to dry overnight before gold sputtering (Edwards Sputter Coater S150B, Crawley, UK) and imaged with a Phillips XL-20 scanning electron microscope (Cambridge, UK).

Fibre diameters and pore sizes were quantified from SEM images of electrospun scaffolds. Each experiment had 3 scaffold samples. Within each 4 images were selected and 10 fibres and 5 pores per image analysed for fibre diameter and pore size, respectively. This gave a total of 120 fibres and 60 pores analysed per scaffold. The software ImageJ (National Institutes of Health) was used for quantification. Pores were identified as areas of void space bounded by fibres on all sides at or near the same depth of field.

### 2.4 Evaluation of mechanical properties

Mechanical properties of wet and dry scaffolds were evaluated. Scaffolds were cut into 0.5 × 1 cm pieces, thickness measured then clamped into a tensiometer (BOSE Electroforce Test Instruments, MN) using a 22 N load cell and a ramp test at a rate of 0.5 mm/s. The first failure point and plateau was used to calculate the ultimate tensile strength (UTS) and the displacement at this point (strain). The initial linear gradient of a plot of stress versus strain was taken as the Young’s modulus (E).

Since the water uptake of our scaffolds occurred gradually the mechanical properties in the wet state were evaluated at days 3, 7, 14 and 21 after the scaffolds were placed in PBS in incubator.

### 2.5 Statistical analysis

Statistical analysis was performed with SPSS v. 17.0. Differences between group means were analysed with Student’s *T* test when the data was normally distributed. Comparisons of more than 2 groups was performed with Kruskal–Wallis test when the data did not demonstrate a normal distribution. Correlation between two continuous variables was assessed by Pearson correlation test. A *p* value of <0.05 was considered statistically significant.

## 3 Results

### 3.1 Effect of AA and A2P on collagen production of fibroblasts in 2D

**Fig. 3:**
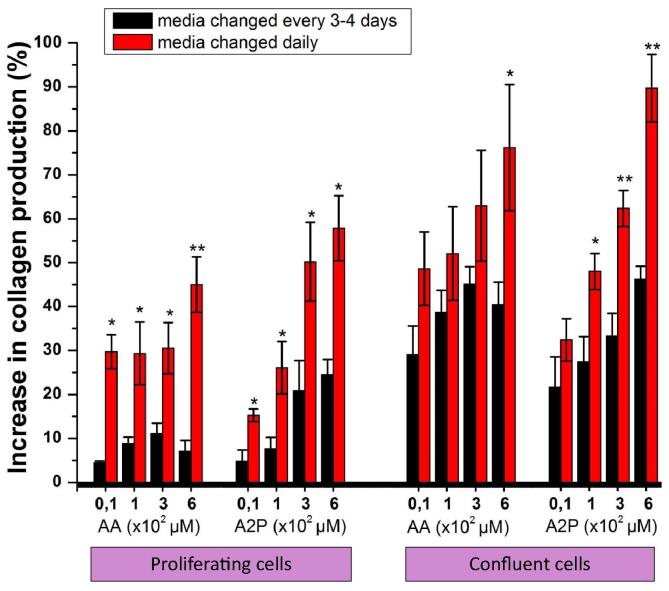
Comparison of total collagen production of proliferating and confluent cultures fibroblasts that were supplemented with AA or A2P either daily or once every 3–4 days (results are at day 14 of culture for proliferating and at day 7 for confluent cultures, ^∗^*p* < 0.05 and ^∗∗^*p* < 0.005, compared to every 3–4 days group).

Daily supplementation of proliferating fibroblasts with 10, 100, 300 and 600 μM concentrations of either AA or A2P resulted in a dose dependent increase in collagen production up to a maximum of 100% by 14 days of culture. Compared to supplementation every 3–4 days, which resulted in a maximum of 50% increase in total collagen production, daily supplementation of AA at a concentration of 600 μM and daily supplementation of A2P at concentrations of 100, 300 and 600 μM resulted in significantly more collagen production (Fig. 3). The same experimental design was then tested for confluent cultures of fibroblasts and a dose dependent increase in collagen production up to a maximum of 60% at 7 days of culture was observed with a significantly more collagen production at all concentrations in the daily supplemented groups (Fig. 3).

### 3.2 Encapsulation efficiency and release of AA and A2P from emulsion electrospun scaffolds

**Fig. 4:**
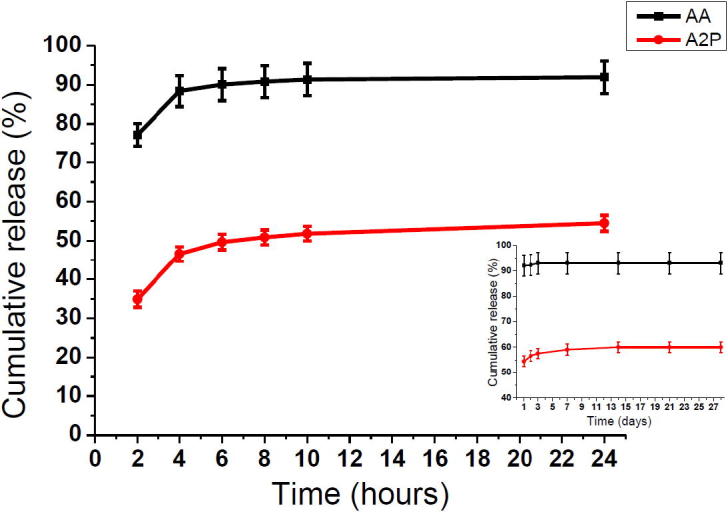
Cumulative release of ascorbic acid (AA) and A2P (Ascorbate-2-Phosphate) from scaffolds into distiled water (dH_2_O) and PBS, respectively. Inset shows release over 28 days [*n* = 6 for each time point, error bars represent standard error of mean].

The encapsulation efficiencies for AA and A2P were calculated to be 46.8% (±6.45) and 44.06% (±7.14). The cumulative release of AA and A2P over 28 days based on the actual drug content are shown in Fig. 4. A burst release of 77% and 34% within 2 h were observed for AA and A2P, respectively. Approximately 90% of AA was released by 10 h and further AA was not detectable after 72 h in dH_2_O. In the case of A2P, 60% of the drug was gradually released over 14 days. Our inability to detect the missing 10% might be due to the oxidation of some of this. Alternatively this small amount remains within the scaffold.

### 3.3 Characterisation of scaffolds

#### 3.3.1 SEM

**Fig. 5:**
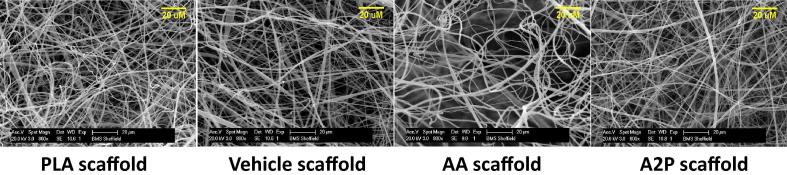
Representative images of scaffolds under scanning electron microscope.

SEM images of the scaffolds are shown in Fig. 5 and these showed there was no significant difference between the AA, A2P, Vehicle and PLA scaffolds in terms of fibre diameter and pore size. The average fibre diameters and pore sizes of AA, A2P, Vehicle and PLA scaffolds were 0.99 (±0.60); 1.04 (±0.56); 1.11 (±0.63) and 1.06 (±0.72) μm and 5.66 (±3.18); 5.76 (±2.76); 5.47 (±3.52) and 5.65 (±4.61) μm, respectively.

#### 3.3.2 Water uptake

**Fig. 6:**
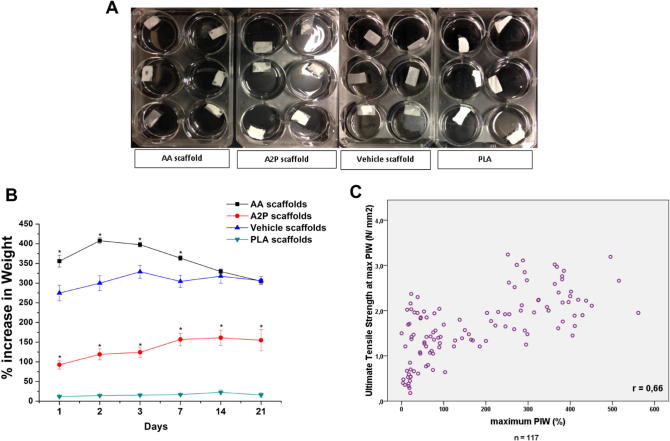
(A) Representative images of scaffolds at day 1 of water uptake experiment, partial wetting of emulsion electrospun (AA, A2P and Vehicle scaffolds) scaffolds can be seen and PLA scaffolds are not wet at all, (B) percent increase in weight of the emulsion electrospun scaffolds upon incubation in PBS over days (^∗^*p* < 0.005 compared to vehicle scaffolds) and (C) correlation of percent increase in weight (PIW) and ultimate tensile strength.

All the emulsion electrospun scaffolds absorbed water gradually over several days (Fig. 6A). The water uptake of AA, A2P and Vehicle scaffolds was significantly higher than that of pure electrospun PLA scaffolds. The water uptake (% increase in weight) of scaffolds containing AA were significantly higher in the first 7 days compared to vehicle scaffolds [355.84 (±140.36)% vs 274.84 (±108.21)% at day 1, *p* = 0.005; 407.69 (±62.38)% vs 299.76 (±92.2)% at day 2, *p* = 0.001; 397.74 (±53.3)% vs 328.76 (±80.31)% at day 3, *p* = 0.001 and 363.64 (±45.32)% vs 304.31 (±65.59)% at day 7, *p* = 0.001] whereas those of A2P containing scaffolds were significantly lower at all-time points compared to vehicle scaffolds [161, 1 (±116.36)% vs 317.55 (±62.25)% at day 14, *p* = 0.001] (Fig. 6B). We also noted that the scaffolds became stronger they more water they absorbed, demonstrated by the correlation between the percentage increase in weight (PIW) and UTS at maximum PIW (Pearson *r* = 0.66, *p* < 0.05) (Fig. 6C).

The effect of different concentrations of AA and A2P on water uptake was also evaluated. No dose dependent change in the water uptake behaviour of the scaffolds were observed in AA scaffolds (mean% increase in weight being 350.52 [±192.54], 325.41 [±191.72] and 321.06 [±186.65] for 0.0001, 0.001 and 0.01 g of AA per gram of PLA concentrations, respectively; Kruskal–Wallis Chi square = 1.40, *p* = 0.49) whereas the medium concentration of A2P scaffolds were slightly more hydrophilic (mean% increase in weight being 72.84 [±64.24], 174.02 [±153.54] and 79.46 [±84.71] for 0.0001, 0.001 and 0.01 g of A2P per gram of PLA concentrations, respectively; Kruskal–Wallis Chi square = 6.69, *p* = 0.03) (Data not shown).

#### 3.3.3 Mechanical properties

**Fig. 7:**
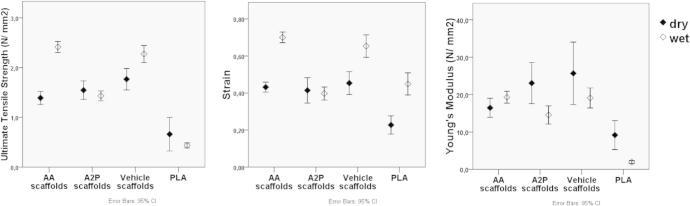
Dry and wet mechanical properties (The values in the y axis are mean values of days 3, 7, 14 and 21; error bars are 95% CI; *n* = 61 and *n* = 240 for dry and wet scaffolds, respectively).

In their dry state, all AA, A2P and Vehicle scaffolds had approximately 2 times higher UTS, strain and YM values compared to pure PLA (Fig. 7). No major differences were observed between AA, A2P and Vehicle scaffolds when dry. When wet the UTS and strain values of AA and Vehicle scaffolds further increased, whereas the Young’s modulus were variable. The mechanical properties of A2P scaffolds did not change significantly when wet compared to when dry (Fig. 7). The UTS of AA scaffolds were significantly higher than Vehicle scaffolds only at day 7 (Table 1).

There was a statistically significant correlation between the percentage increase in weight and the UTS of wet scaffolds at day 3, 7, 14 and 21 (Pearson *r* = 0.64 [*p* < 0.001], 0.78 [*p* < 0.001], 0.67 [*p* < 0.001] and 0.85 [*p* < 0.001], respectively), the strongest correlation was observed at day 21. The collected data is shown in Fig. 6C.

### 3.4 Cell viability and collagen production of fibroblasts grown on scaffolds

**Fig. 8:**
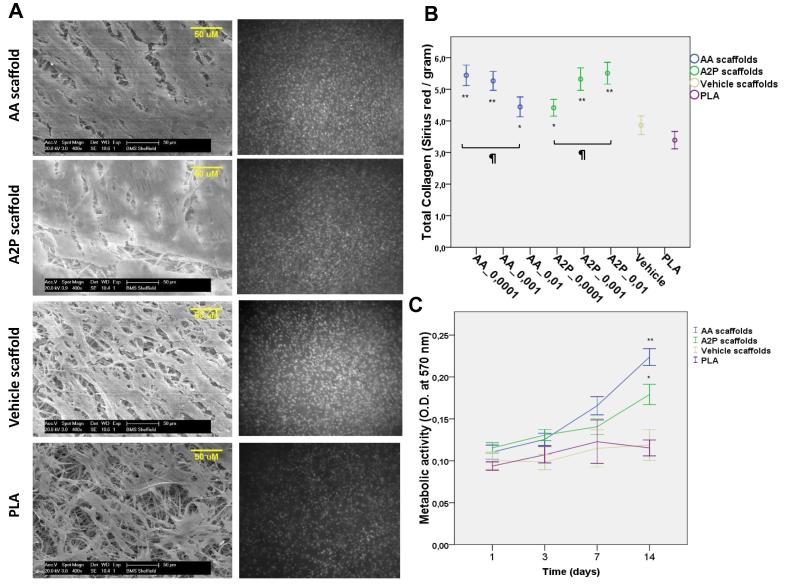
Production of ECM by human dermal fibroblasts grown on AA, A2P, Vehicle and PLA scaffolds for 14 days (A) Representative SEM images and corresponding DAPI staining of the cell nuclei of fibroblasts, (B) total collagen production at day 14 as measured by Sirius Red staining [numbers after letters show grams of AA or A2P per gram of PLA] and (C) metabolic activity over 14 days [*n* = 94; error bars show SEM; ^∗∗^*p* < 0.005 and ^∗^*p* < 0.05 compared to Vehicle scaffolds; ^¶^*p* > 0.05].

The SEM images of scaffolds on which fibroblasts were grown for 14 days, showed continuous coverage of the scaffold surface by fibroblasts and extracellular matrix which was more intense in AA and A2P containing scaffolds (Fig. 8A). A quantitative analysis of collagen production by Sirius Red staining showed significantly more collagen production on all concentration of AA and A2P scaffolds compared to Vehicle scaffolds (Fig. 8B). The mean corrected absorbance values (Sirius Red stain/gram of PLA) were 5.44 (±0.50); 5.26 (±0.46); 4.44 (±0.49) and 4.41 (±0.41); 5.32 (±0.55); 5.50 (±0.51) for AA and A2P scaffolds with concentrations of 0.0001, 0.001 and 0.01 g of either AA or A2P per gram of PLA, respectively. Corresponding values for Vehicle and PLA scaffolds were 3.86 (±0.43) and 3.39 (±0.43), respectively. Although lower concentrations of AA and higher concentrations of A2P seemed to work better, no single concentration of either AA or A2P proved to be significantly different than other concentrations (Fig. 8B). Also metabolic activity of human dermal fibroblasts grown on AA and A2P scaffolds was increased compared to Vehicle scaffolds, becoming statistically significant after 14 days of culture (Fig. 8C).

## 4 Discussion

There is a need for new approaches to support weakened tissues of the pelvic floor for women suffering from POP and SUI. The aim of this study was to develop and evaluate the use of ascorbic acid containing scaffolds as an approach to stimulate new tissue production for pelvic floor repair while maintaining desirable mechanical properties.

The main findings were that it was possible to introduce either AA or A2P into biodegradable scaffolds without compromising mechanical properties, to measure the release of both compounds into the media and finally that both compounds stimulated collagen production in fibroblasts cultured on the scaffolds.

AA is well documented for its effect in stimulating collagen production via several routes and there are various analogues of it, of which the one used most often in cell culture is A2P because of its reported greater stability. Accordingly this study began by evaluating the comparative effectiveness of AA and A2P on collagen production of cultured fibroblasts in 2D as we were unable to find any long-term comparative data on these two in the literature. The 2D comparison studies were done looking at replacing AA and A2P either daily to mimic the continuous release state that would overcome the major disadvantages of instability of AA or every few days. Our findings showed that both AA and A2P increased collagen production of fibroblasts to a similar extent in a dose dependent manner.

When AA was given daily (as opposed to being replaced every 3–4 days as would normally occur in routine culture of cells) then AA was as effective as A2P. Further, there was stimulation of collagen production in both proliferating and confluent fibroblasts and the concentration which became toxic to cells was in excess of 600 μM which confirms previous studies [16]. This data essentially confirms several previous studies demonstrating increased collagen production when media is supplemented with different derivatives of AA [16], [19], [33], [34], [35], [36]. Comparing both AA and A2P under identical conditions with different replacement schedules (daily versus every 3–4 days) and in proliferative versus confluent fibroblasts allows us to conclude that both AA and A2P are good candidates for application in a tissue engineering scaffold.

With emulsion electrospinning we were able to encapsulate nearly 50% of AA and A2P within the PLA micro/nano fibres. The initial burst release of AA was higher compared to A2P. Encapsulation efficiency and burst release are two related parameters showing how effectively the drug is captured within the polymer. Factors such as physical and chemical properties of the polymer and the drug, polymer–drug interactions and the emulsion conditions are known to affect these parameters [37]. The use of electrospun fibres for drug encapsulation and delivery is increasing in popularity and it can be achieved by emulsion electrospinning and co-axial electrospinning techniques [38]. Effective drug encapsulation (between 17% and 90%) [39], [40], [41], [42] and variable burst release rates (between 20% and 73%) with several drug–polymer combinations [31], [41], [43] with use of these techniques have been reported. The difference in the encapsulation and release pattern of AA and A2P can be explained by the difference in their chemical structure. Also the use of distiled water for AA release might have an impact on these parameters as it is different from PBS in terms of pH and ionic strength. However it was impossible for us to carry out a release experiment of AA in PBS because the rapidly oxidising AA reacted with the metal ions in PBS as soon as it was released. On the other hand when AA was released in dH_2_O it was oxidising with the oxygen in the air however this did not happen immediately (after 6 h in our experiments) allowing us to carry out the experiment at 2 hourly intervals. The relative stability of AA at pH: 7 in the absence of metal ions was previously demonstrated [44]. Conclusively, we are demonstrating a reasonable encapsulation efficiency of both AA and A2P and as expected A2P demonstrates a more sustained release pattern compared to the release of AA from the more hydrophilic AA scaffolds.

The next stage in the study was to culture fibroblasts on the scaffolds and look at the effect of the ascorbic acid releasing scaffolds on cell viability and collagen production. We observed significant increase in collagen production in these cells when grown on AA and A2P scaffolds.

Structurally we found that using the surfactant, Span80, to produce emulsions within the scaffolds for AA, A2P and vehicle controls did not affect the gross structure of the scaffolds as seen in SEM images. However, there were effects of the surfactant and of AA and A2P on the mechanical properties and water absorptive capacity of the scaffolds. Surprisingly in the dry state all emulsion electrospun scaffolds were stronger and more elastic than non-loaded PLA scaffolds. These emulsion electrospun scaffolds absorbed water gradually over several days and there was a positive correlation between water uptake and the ultimate tensile strength. This is almost certainly explained by the plasticizing effect of water on PLA scaffolds [45]. Other surfactants such as polyethylene glycol and glucose monoesters, has previously been reported to act as a plasticisers [46]. AA scaffolds were more hydrophilic in the first 7 days (Fig. 6B) and slightly but significantly stronger by day 7 (Table 1). This taken together with the release of AA from the scaffolds, suggests a contribution of AA to hydrophilicity and strength of these scaffolds.

In other studies both AA and A2P have been shown to increase ECM production of cells cultured on scaffolds in 3D [47], [48], [49], [50] with A2P being the more frequently used because of its greater stability. To the best of our knowledge this is the first study to directly compare scaffolds releasing AA and A2P for their effects on cells grown on them. Interestingly the 3D results showed increased collagen production with increasing levels of A2P, as expected but with the AA containing scaffolds the collagen production did not increase with higher concentrations of AA. This is almost certainly explained by the toxicity of AA at high concentrations *in vitro* that might have occurred in the 3D microenvironment within the fibres of the scaffold.

In conclusion, with respect to scaffold production this data contributes to the literature by showing how incorporation of a detergent can act as a plasticiser with changes in the mechanical properties of the scaffolds. The incorporation of AA and A2P did not weaken the scaffolds-indeed almost certainly because of the incorporation of the detergent Span, these scaffolds were both stronger and more elastic.

With respect to the usefulness of these materials for clinical use we suggest that scaffolds releasing ascorbic acid could help the integration of scaffolds into the pelvic floor by stimulating production of adjacent tissues in the region. Also these scaffolds could be introduced with cells in a tissue engineering approach and again the scaffolds may stimulate new tissue production by the cells on the scaffolds.

In developing materials for surgical treatment of POP and SUI it is important to make materials that are biomechanically compatible with the anatomical site of implantation [51], [52]. The introduction of an ascorbic acid releasing scaffold that will stimulate new collagen production and whose mechanical properties are not compromised by the introduction of ascorbic acid is, we suggest, a significant contribution to developing new materials for this challenging site.

## 5 Conclusion

It is possible to stimulate new collagen production by incorporation of both AA and A2P as emulsions into biocompatible, biodegradable electrospun PLA scaffolds. Incorporation of these forms of ascorbic acid actually increases the strength and elasticity of scaffolds and may provide valuable new biomaterials for the applications of the pelvic floor repair.

## Figures and Tables

**Table 1:** Mechanical properties at each time point [mean (±SD) values for UTS (Ultimate Tensile Strength), Strain and YM (Young’s modulus)] (^∗∗^*p* < 0.005, ^∗^*p* < 0.05 compared to vehicle scaffolds; ^++^*p* < 0.005 ^+^*p* < 0.05 compared to PLA scaffolds).

	DRY (*n* = 61)			WET_day 3 (*n* = 48)			WET_day 7 (*n* = 48)			WET_day 14 (*n* = 48)			WET_day 21 (*n* = 48)		
	UTS	Strain	YM	UTS	Strain	YM	UTS	Strain	YM	UTS	Strain	YM	UTS	Strain	YM
AA scaffolds	1.39^++,∗∗^	0.43^++^	16.46^++,∗∗^	2.37^++^	0.66^++^	20.07^++^	2.93^++,∗∗^	0.77^++^	24.06^++^	2.36^++^	0.73^++^	13.57^++^	2.26**^++^**	0.59**^++^**	21.78**^++,^**^∗^
(SD)	(0.30)	(0.06)	(5.99)	(0.45)	(0.15)	(7.81)	(0.55)	(0.08)	(8.06)	(0.44)	(0.12)	(4.70)	(0.39)	(0.17)	(5.60)
A2P scaffolds	1.54**^++^**	0.41**^++^**	23.08**^++^**	1.49**^++,^**^∗∗^	0.48	14.76**^++^**	1.28**^++,^**^∗∗^	0.44^∗∗^	9.62**^+,^**^∗^	1.52**^++,^**^∗∗^	0.37^∗∗^	15.49**^++^**	1.27^++,∗∗^	0.31**^+,^**^∗∗^	16.56**^++,^**^∗^
(SD)	(0.41)	(0.15)	(12.07)	(0.38)	(0.48)	(10.81)	(0.48)	(0.09)	(7.03)	(0.37)	(0.18)	(10.29)	(0.46)	(0.15)	(12.75)
Vehicle scaffolds	1.76**^++^**	0.45**^++^**	25.68**^++^**	2.36**^++^**	0.58**^++^**	20.97**^++^**	2.10**^++^**	0.71**^+^**	16.93**^++^**	2.23**^++^**	0.66**^+^**	14.18**^++^**	2.45**^++^**	0.47**^++^**	27.08**^++^**
(SD)	(0.25)	(0.07)	(9.03)	(0.34)	(0.17)	(6.42)	(0.57)	(0.12)	(6.51)	(0.66)	(0.17)	(5.54)	(0.35)	(0.07)	(4.56)
PLA	0.66	0.22	9.17	0.71	0.40	3.56	0.34	0.45	1.80	0.54	0.36	2.75	0.30	0.40	1.70
(SD)	(0.36)	(0.05)	(4.20)	(0.23)	(0.11)	(2.12)	(0.10)	(0.16)	(0.73)	(0.09)	(0.15)	(1.86)	(0.04)	(0.05)	(0.79)

